# An Ear Replaced after Complete Severance

**Published:** 1898-06-11

**Authors:** 


### AN EAR REPLACED AFTER COMPLETE SEVERANCE.

The reapplication of a part which has been completely severed from the body is a thing which has often been done, but very seldom with much success, except when the part removed has been of very small dimensions. Much interest, then, attaches to a case reported in the *Lancet* by Dr. W. J. Brown, of Chester-le-Street, in which the external ear was completely bitten off by a horse, and yet, after careful reapplication, in great part survived. The patient was a boy, aged 14. "He presented a hideous appearance, the greater portion of the pinna, together with a semi-circular flap of an inch radius from behind the ear, having been bitten off, leaving only the tragus, with a quarter of an inch each of the helix and the lobule." Although the case looked so hopeless, Dr. Brown determined to make an attempt to replace the ear if it could be found. After about ten minutes' search it was brought, having been discovered near the horse in the stable yard. "It was of a dirty drab colour, and the posterior flap was curled up in a roll." Having no instruments with him (the affair had happened at an auction at which the doctor was present), he procured some common needles and thread, and, after washing the ear in warm water, proceeded to sew it in place. Eight sutures were used. A roll of cotton wool was then placed behind the ear to support it, and the ear having been wrapped in cotton wool and covered with a silk handkerchief, the boy was sent home. As soon as possible after this as much of the wool as could be removed without disturbing the wound was cleared away, the surface was dusted with iodoform, and the ear was wrapped in cotton wool and gamgee tissue. After five days the dressings were removed, the ear was washed with a warm solution of perchloride of mercury (one in 1,000), was dusted with iodoform, was wrapped in iodoform gauze, and packed round and covered with cotton wool. After this the dressings were changed at intervals of a few days, and within six weeks of the accident the wound was quite healed. A small portion of the lobe sloughed, but otherwise the boy, as seen in a photograph, shows a very handsome ear. It is worthy of note that although antiseptics were carefully used after the ear was reapplied, none came in contact with the surfaces of adhesion.

